# *LPG2* Gene Duplication in *Leishmania infantum*: A Case for CRISPR-Cas9 Gene Editing

**DOI:** 10.3389/fcimb.2020.00408

**Published:** 2020-08-13

**Authors:** Flávio Henrique Jesus-Santos, Jéssica Lobo-Silva, Pablo Ivan Pereira Ramos, Albert Descoteaux, Jonilson Berlink Lima, Valéria Matos Borges, Leonardo Paiva Farias

**Affiliations:** ^1^Laboratory of Inflammation and Biomarkers, Instituto Gonçalo Moniz, Fundação Oswaldo Cruz (FIOCRUZ), Salvador, Brazil; ^2^Faculdade de Medicina da Bahia, Federal University of Bahia (UFBA), Salvador, Brazil; ^3^Center for Data and Knowledge Integration for Health (CIDACS), Instituto Gonçalo Moniz, Fundação Oswaldo Cruz, Salvador, Brazil; ^4^Institut National de la Recherche Scientifique—Centre Armand-Frappier Santé Biotechnologie, Laval, QC, Canada; ^5^Center of Biological Sciences and Health, Federal University of Western of Bahia (UFOB), Barreiras, Brazil

**Keywords:** GDP-mannose transporter, lipophosphoglycan, *Leishmania infantum*, gene targeting, CRISPR/CAS9

## Abstract

On the surface of the *Leishmania* promastigote, phosphoglycans (PG) such as lipophosphoglycan (LPG), proteophosphoglycan (PPG), free phosphoglycan polymers (PGs), and acid phosphatases (sAP), are dominant and contribute to the invasion and survival of *Leishmania* within the host cell by modulating macrophage signaling and intracellular trafficking. Phosphoglycan synthesis depends on the Golgi GDP-mannose transporter encoded by the *LPG2* gene. Aiming to investigate the role of PG-containing molecules in *Leishmania infantum* infection process, herein we describe the generation and characterization of *L. infantum LPG2*-deficient parasites. This gene was unexpectedly identified as duplicated in the *L. infantum* genome, which impaired gene targeting using the conventional homologous recombination approach. This limitation was circumvented by the use of CRISPR/Cas9 technology. Knockout parasites were selected by agglutination assays using CA7AE antibodies followed by a lectin (RCA 120). Five clones were isolated and molecularly characterized, all revealing the expected edited genome, as well as the complete absence of LPG and PG-containing molecule expression. Finally, the deletion of *LPG2* was found to impair the outcome of infection in human neutrophils, as demonstrated by a pronounced reduction (~83%) in intracellular load compared to wild-type parasite infection. The results obtained herein reinforce the importance of LPG and other PGs as virulence factors in host-parasite interactions.

## Introduction

*Leishmania* promastigotes are coated by a thick glycocalyx consisting of glycoconjugates crucial to parasite pathogenesis. Lipophosphoglycan (LPG), proteophosphoglycan (PPG), and glycophosphatidylinositol lipids (GIPL), as well as the GP63 metalloprotease, comprise the vast majority of these molecules. *Leishmania* also secrete protein-linked phosphoglycans (PG) (e.g., secreted proteophosphoglycan (sPPG) and secreted acid phosphatase (sAP) (reviewed in Guha-Niyogi et al., 2001; Franco et al., 2012; Forestier et al., 2015). In promastigotes, LPG plays an important role in parasite survival inside the sand fly vector, in addition to macrophage infection (Sacks et al., 2000; Balaraman et al., 2005; Moradin and Descoteaux, 2012). Moreover, intracellular survival and the multiplication of amastigotes in macrophages is enhanced by other PG-containing molecules (e.g., PPG and sAP), which are highly expressed on the surface of amastigotes (Gaur et al., 2009).

LPG is organized into four domains: a conserved 1-O-alkyl-2-lyso-phosphatidyl(myo)inositol membrane anchor, a conserved diphosphoheptasaccharide core structure, a polymer that consists of repeating phosphodisaccharide units (phosphoglycan or PG) and carries species-specific side chains, as well as and variable mannose-rich cap structures (reviewed in McConville et al., 1993; Descoteaux and Turco, 1999). The PPGs comprise a heterogeneous family of cell surface and secreted proteins containing Ser-Thr rich regions to which phosphodisaccharide repeating units (Manα1-PO4 residue) (PG) are covalently linked, similarly to the LPG molecule (reviewed in Ilg, 2000b; de Assis et al., 2012). This type of phosphoglycosylation is the most abundant type of protein glycosylation found in *Leishmania*. Investigations focused on the synthesis of LPG and PG-containing molecules (PPG, PGs, sAP) have attracted considerable interest, and several enzymes and transporters involved in this process have been identified either biochemically, genetically, or both (Ryan et al., 1993; Descoteaux et al., 1995, 1998, 2002).

An enzyme critical for the synthesis of LPG and PG-containing molecules is the Golgi GDP-mannose transporter (encoded by *LPG2*) (Descoteaux et al., 1995), which contains up to nine transmembrane domains and presents a TPT domain, which is found in many transporters with affinity for triose phosphate. In *Leishmania*, this gene is required for the addition of disaccharide-phosphate units to lipophosphoglycan and related glycoconjugates (Ma et al., 1997; Hong et al., 2000; Segawa et al., 2005).

Over the past decades, the development of *Leishmania* mutants deficient in LPG or other PG-containing molecules has provided researchers with powerful tools to analyze the function of these structures/molecules (McNeely et al., 1990; Descoteaux et al., 1995; Butcher et al., 1996). *L. major* and *L. donovani* Δ*lpg2* mutants failed to survive in the midgut of the sand fly vector and were unable to establish infection in macrophages. In an animal infection model, *L. major* parasites were found to exhibit persistence without causing pathological manifestations, with parasites persisting at low levels throughout the life of the infected animals (Spath et al., 2003b). However, whether LPG and PGs are necessary for parasite survival in all *Leishmania* species has been contested based on *L. mexicana* studies. Although Δ*lpg1* and Δ*lpg2* parasites of this species exhibit complement sensitivity, no decreases in infectivity were observed *in vitro* in macrophages or *in vivo* in mice (Ilg, 2000a; Ilg et al., 2001; Gaur et al., 2009). Our group recently generated an LPG-deficient mutant of *L. infantum* through the deletion of the putative galactofuranosyl transferase gene (*LPG1)* involved in the synthesis of the LPG glycan core. Phenotypically, this deletion impaired the outcome of infection in murine bone marrow-derived macrophages, likely due to the activation of the iNOS promoter in an NF-κB-dependent manner (Lazaro-Souza et al., 2018). While these parasites expressed a truncated LPG molecule lacking the PG domain, they were still able to assemble and secrete other PG-containing molecules (e.g., PPG, sAP, and other PGs) (Dermine et al., 2000; Spath et al., 2000).

In an effort to investigate the role of PG-containing molecules in the *Leishmania infantum* infection process, we applied gene targeting by homologous recombination and also CRISPR/Cas9 technology to generate an *L. infantum* mutant lacking the Golgi GDP-mannose transporter gene (Δ*lpg2*). Our results demonstrate both the value and the caveats of using of both systems for genome editing. The generated mutant produced distinct infection outcomes in comparison to WT parasites, and can therefore be usefully applied to investigate the role of *L. infantum* PG-containing molecules in host-parasite interactions.

## Methods

### Ethics Statement

This study was approved by the Institutional Review Board of Human Ethical Research Committee of Fundação Oswaldo Cruz-Bahia, under number 100/2006.

### Parasite Cultures

*Leishmania infantum* Ba262 (MCAN/BR/89/BA262) promastigotes were cultured in HOMEM medium supplemented with 10% inactivated Fetal Bovine Serum (FBS), 100 U/mL penicillin, 100 μg/mL streptomycin and 2 mM L-glutamine in 25 cm^2^ flasks at 25°C until late log-phase. The number of promastigotes was determined by counting in a Neubauer chamber.

### *LPG2* Gene Targeting by Homologous Recombination

Initially, we attempted to obtain an *L. infantum* LPG-deficient mutant (Δ*lpg2*) by homologous recombination, using a previously described strategy (Scianimanico et al., 1999) with modifications. Briefly, the gene sequences from the resistance markers Hygromycin and Neomycin were amplified by PCR using specific oligonucleotides (Supplementary Table 1) and ligated in the pUC18 vector (Sigma, USA), thusly generating the pUC18-Hyg and pUC18-Neo constructs. Next, the 5' and 3' UTR *LPG2* gene regions, consisting of 838 bp and 858 bp fragments respectively, were amplified by PCR from *L. infantum* Ba262 (MCAN/BR/89/BA262) genomic DNA using a specific oligonucleotide design based on the 19 kb genomic contig (GenBank accession CACT01000040.1), which was predicted to encode the *LPG2* gene (LinJ_34_4290) (Supplementary Table 1). The respective fragments were then digested with *Eco*RV/*Kpn*I and *Xba*I/*Hind* III enzymes, and ligated in the pUC18-Hyg and pUC18-Neo constructs. The final constructs were sequenced for confirmation and designated p*LPG2*-Hyg and p*LPG2*-Neo. Log-phase WT *L. infantum* promastigotes were electroporated in two steps with 10 μg of purified fragments (p*LPG2*-Neo and p*LPG2*-Hyg) using 0.4 cm cuvettes in a Gene Pulser II (BIO-RAD) under electroporation conditions based on a previously described high voltage protocol (Robinson and Beverley, 2003). Briefly, two pulses were applied at 10-s intervals (25 μF, 1,500 V). Following electroporation, promastigotes were incubated for 24 h at 25°C in drug-free medium, and the neo-resistant parasites were subsequently selected in the presence of 70 μg/mL G418 for 22 days at 25°C. The resistant parasites (*lpg2*^+/−^) were isolated and submitted to a second round of electroporation using the p*LPG2*-Hyg fragment to disrupt the second allele of the *LPG2* gene. Double knockout (KO) Δ*lpg2* parasites were then selected in the presence of both 70 μg/mL G418 and 50 μg/mL Hygromycin B for 22 days at 25°C. The absence of the *LPG2* gene in the resulting double drug-resistant promastigotes was verified by PCR following DNA extraction using “NucleoSpin tissue” kit (Macherey Nagel).

### gRNA Design and Cloning Into pLdCN

The present procedures involving the CRISPR/Cas9 system were based on pioneering work previously performed by Zhang and Matlashewski (2015) and Zhang et al. (2017) with minimal modifications. Two gRNA-targeting sequences and the respective oligonucleotide donors (oligodonors) were selected using the gRNA designer tool (http://grna.ctegd.uga.edu/) and then manually inspected (Supplementary Table 1). The complementary guide sequence oligonucleotides were first phosphorylated in T4 DNA ligase buffer with T4 polynucleotide kinase and then annealed in a thermocycler (MJ Research: PTC-200, DNA Engine) (program: 95°C for 5 min, ramp to 25°C at a rate of −0.1°C/s). The annealed guide sequence was then cloned into the gRNA and Cas9 coexpression vector (pLdCN) (Addgene plasmid #84290; http://n2t.net/addgene:84290; RRID:Addgene_84290) previously digested with *Bbs*I.

### Parasite Transfection (CRISPR/Cas9)

To obtain *L. infantum* parasites expressing Cas9, log-phase WT *L. infantum* promastigotes were electroporated with purified pLdCN (gRNA440 and gRNA516) under the same conditions described in the section above (*LPG2* gene targeting by homologous recombination). After selection with G418 (70 μg/mL), four rounds of transfection were performed using 100 μM single-stranded oligodonors at 3-day intervals (Zhang et al., 2017).

### *LPG2* Knockout Selection and Limiting Dilution Assay

After transfection with the oligodonors, the putative Δ*lpg2* parasites were selected by consecutive rounds of agglutination with the CA7AE monoclonal IgM antibody (MediMabs) (1:2,000 dilution), which recognizes the Gal(β1,4)Man(α1-PO4) repeating units contained in LPG and PG-containing molecules (Descoteaux et al., 1998). Briefly, parasites were incubated for 2 h with the antibody and then centrifuged at 100 × g for 7 min to remove any agglutinated promastigotes. The supernatant was then transferred to a new tube and centrifuged at 1,300 × g for 7 min to sediment non-agglutinated parasites (theoretically Δ*lpg2*). Subsequently, these non-agglutinated parasites were incubated in fresh culture medium containing CA7AE (1:2,000) for 3 days, followed by five rounds of the above-described agglutination steps. As a further selection step, non-agglutinated parasites were selected with 100 μg/ml of Ricin 120 (Vector Laboratories, USA) as described in King and Turco (1988). After selecting the Δ*lpg2* parasites, the pLdCN vector, as well as Cas9 protein expression, were eliminated by growing parasites in the absence of the G418 marker, which was achieved after 5–6 passages. Finally, a limiting dilution assay was performed for clonal isolation using 96-well plates with culture medium containing 100 μg/mL of ricin, incubated for 3 weeks at 25°C with medium supplementation when necessary. To calculate doubling times, cells were seeded at 10^5^ cells/ml and density was measured every 24 h. The average doubling time, from day zero until reaching stationary phase, was calculated by (24/(log2(t2/t1)))/number of days evaluated (Beneke et al., 2019). Subsequently, genomic DNA was extracted from the last dilution in which parasite growth was observed.

### Sequencing Analysis

The sequence corresponding to the 5'UTR-Neo/Hyg/*LPG2*-3'UTR genomic DNA region was amplified with specific oligonucleotides (Supplementary Table 1) by PCR. The PCR products were purified on agarose gels (Wizard® SV Gel and PCR Clean-Up System) and sequenced using the Gonçalo Moniz Institute sequencing platform.

### Western Blotting

Late log-phase promastigotes at a concentration of 2 × 10^7^ cells were centrifuged and resuspended in 100 μl of RIPA Lysis Buffer (150 mM NaCl, 0.5 M EDTA, pH 8.0, 1 M Tris, pH 8.0, 1%NP-40, 1% sodium deoxycholate, 0.1% SDS and dH_2_O) containing a protease inhibitor (Sigma, cat. No. P-2,714). The extract was incubated on ice and vortexed for approximately 5 min. The lysated material was separated by SDS-PAGE on a 12% polyacrylamide gel and Western blotting was performed as previously described (Lazaro-Souza et al., 2018).

### T7 Endonuclease I Assay

Briefly, genomic DNA from *L. infantum* WT and Δ*lpg* clones was extracted using a “NucleoSpin tissue” kit (Macherey Nagel). The sequences corresponding to the *LPG2* and Δ*lpg2* genes were amplified by PCR with specific oligonucleotides (Supplementary Table 1) and purified from agarose gels using the Wizard® SV Gel and PCR Clean-Up System. Subsequently, 200 ng of the purified PCR product was denatured and re-annealed at a final volume of 19 μl in 1 × NEBuffer2 (NEB) using a thermocycler in accordance with the following protocol: 95°C for 5 min; ramp to 85°C at a rate of −2°C/s; ramp to 25°C at a rate of −1°C/s; hold at 4°C. The PCR products were then treated with T7 E1 enzyme (NEB) at 37°C for 15 min at a reaction volume of 20 μl. An aliquot (10 μl) of the reaction was subsequently analyzed on a 1% agarose gel.

### Confocal Immunofluorescence Microscopy

Late log-phase promastigotes were adhered on Poly-L-Lysine-coated glass coverslips (BD Biosciences, San Jose, CA) by centrifugation, fixed with 4% paraformaldehyde for 20 min and simultaneously blocked and permeabilized for 20 min using a solution containing 0.1% Triton X-100, 1% BSA, 6% non-fat dry milk, 20% goat serum and 50% FBS. The distribution of LPG and other PGs containing the Gal(β1,4)Man(α1-PO4) repeating unit epitope was visualized using the CA7AE mouse monoclonal antibody (MediMabs, 1:2,000) after a 2 h incubation period, followed by an additional 30 min incubation with Alexa Fluor 488 goat anti-mouse IgM (Molecular Probes) at 1:500. Parasite nuclei were stained with DAPI (Molecular Probes) at 1:17,000. All steps were performed at room temperature. The coverslips were then mounted in Fluoromount-G (Interscience) and sealed with nail polish. Parasites were observed under a Plan APOCHROMAT 63 × oil-immersion DIC 1.4 NA objective on a Leica SP8 confocal microscope in reflective mode, using a 488 nm laser, with an LP505 filter.

### Infection Assay

Human blood was obtained from volunteers at the Bahia Foundation of Hematology and Hemotherapy (HEMOBA). Human neutrophils were isolated by gradient separation in polymorphonuclear medium (PMN) according to the manufacturer's instructions (Axis-ShieldPoc AS, Oslo, Norway). Neutrophils were collected and washed three times with saline at 4°C for 10 min at 300 x g. Neutrophils were plated with RPMI-1640 medium, supplemented with 1% Nutridoma-SP, 2 mM L-glutamine, 100 U/ml penicillin and 100 μg/ml of streptomycin. Cells were infected with stationary WT or Δ*lpg2* promastigotes at an MOI of 10 for 3 h at 37°C under 5% CO_2_. The intracellular load of *L. infantum* was then evaluated in neutrophils by assessing parasite viability as previously described (Quintela-Carvalho et al., 2017). Briefly, after 3 h of incubation, infected neutrophils were centrifuged at 300 × g for 10 min at 4°C, the supernatant containing non-internalized promastigotes was discarded and replaced by 250 μl of HOMEM medium. After a final incubation at 23°C for 24 h, the number of viable proliferating extracellular promastigotes was counted in a Neubauer chamber.

### Statistical Analysis

Diferences in growth curves were analyzed using Area under the Curve (AUC) analysis. Neutrophil infection assays were performed twice from at least five donors, data are presented as means and SE (standard error) of representative experiments. Wild-type and knockout groups were compared using the Student's *t-*test. Differences were considered statistically significant when *p* ≤ 0.05. All figures and statistical analyses were performed using PRISM version 5.02 software (GraphPad, San Diego, CA).

## Results

### *LPG2* Is Duplicated in *L. infantum*

**Figure 1:**
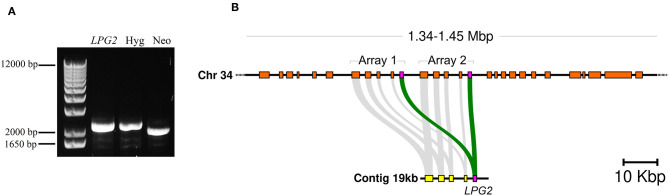
Evidence of *LPG2* duplication in *L. infantum*. **(A)** Genomic DNA from *L. infantum* Hyg/NeoR strain was amplified by PCR using specific oligonucleotides (Supplementary Table 1) to verify *LPG2* gene targeting by homologous recombination. **(B)** Comparison of the genomic contexts where *LPG2* (magenta box) was originally located (in an unplaced 19 kbp contig shown at the bottom) to the recent resequencing and reassembly of the *L. infantum* JPCM5 genome (Gonzalez-de la Fuente et al., 2017), where the duplication of the *LPG2* gene array is evidenced in chromosome 34 (top). Gray connecting segments indicate sequence conservation, and the coordinates at the top refer to the novel chromosome 34 assembly.

After selection of double drug-resistant (Neo/Hyg) parasites, the absence of the *LPG2* gene was assessed by PCR. Electrophoresis results confirmed the expected occurrence of homologous recombination and the successful integration of the resistance markers (Neo and Hyg). However, surprisingly, the coding region of the *LPG2* gene was still detected following amplification (Figure 1A).

This effectively indicated that either homologous recombination did not occur, or that the *LPG2* gene is duplicated in this species. No available evidence suggested the latter possibility, since the *L. infantum* JPCM5 genome assembly published at the time of the initial recombination experiments showed *LPG2* as a single-copy gene located in an unplaced 19 kb contig (GenBank accession CACT01000040.1). However, a more recent resequencing of the JPCM5 genome, which employed a hybrid sequencing approach with PacBio long reads and Illumina short reads, yielded a more robust assembly containing 36 scaffolds accounting for the 36 chromosomes present in this species (Gonzalez-de la Fuente et al., 2017) (EMBL accession GCA_900500625.1). BLAST searches of the 19 kb contig as a query against this novel assembly revealed that the entire contig, including the *LPG2* gene, was duplicated in a tandem array in chromosome 34 of *L. infantum* JPCM5 (Figure 1B and Supplementary Figure 1A). This finding highlights a limitation associated with the obtainment of knockout parasites using conventional homologous recombination procedures.

### Generation of *L. infantum* Expressing Cas9

**Figure 2:**
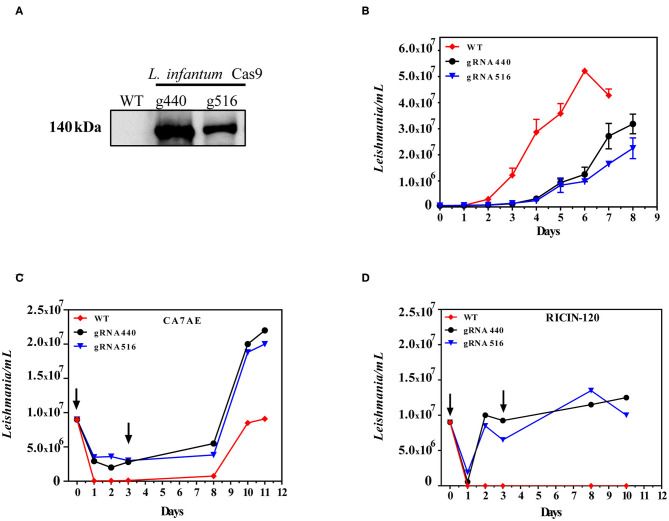
Generation of *LPG2* knockout using CRISPR/Cas9. **(A)** Western blot analysis of *L. infantum* promastigotes expressing Cas9 (gRNA440 and gRNA516). **(B)** Growth curves of *L. infantum* wild-type (WT) and *L. infantum*-Cas9 (gRNA440 and gRNA516) promastigotes. **(C)** Agglutination assay using the CA7AE monoclonal antibody and associated growth curves. **(D)** Agglutination assay using Ricin 120 lectin and associated growth curves, demonstrating the selection of Δ*lpg2*. The arrows indicate the time points where antibody (CA7AE) or lectin (Ricin-120) were added to the cultures, exemplifying the typical agglutination results observed.

As a first step to perform *LPG2* gene editing, we developed *L. infantum* parasites expressing the Cas9 enzyme. WT parasites were transfected with the pLdCN plasmid and selected in G418 culture medium to generate the strain constitutively expressing the Cas9 nuclease. The expression of the Cas9 enzyme was confirmed in G418-resistant parasites by Western blotting (Figure 2A). The growth curves of promastigotes revealed that the expression of Cas9-gRNAs influenced parasite growth. A delay in the replication capability of the Cas9-gRNA expressing parasites was observed in comparison to WT. In addition, the former reached stationary phase by day 6, with a cell density of ~5 × 10^7^, while the latter reached this phase 2 days later, with a cell density of ~3 × 10^7^ (Figure 2B). Sequencing data revealed no evidence of *LPG2* gene editing at this initial stage (data not shown). Next, *L infantum* expressing Cas9 were submitted to four rounds of transfection using single-stranded oligodonors to improve *LPG2* gene disruption and knockouts were subsequently selected.

### Selection of *L. infantum Δlpg2*

Five rounds of selection using the CA7AE antibody were unexpectedly unsuccessful in completely removing WT parasites by agglutination (Figure 2C). This was circumvented through the use of Ricin 120, after which complete depletion of WT *L. infantum* and the selection of Δ*lpg2* was observed (Figure 2D).

### Characterization of *L. infantum Δlpg2*

**Figure 3:**
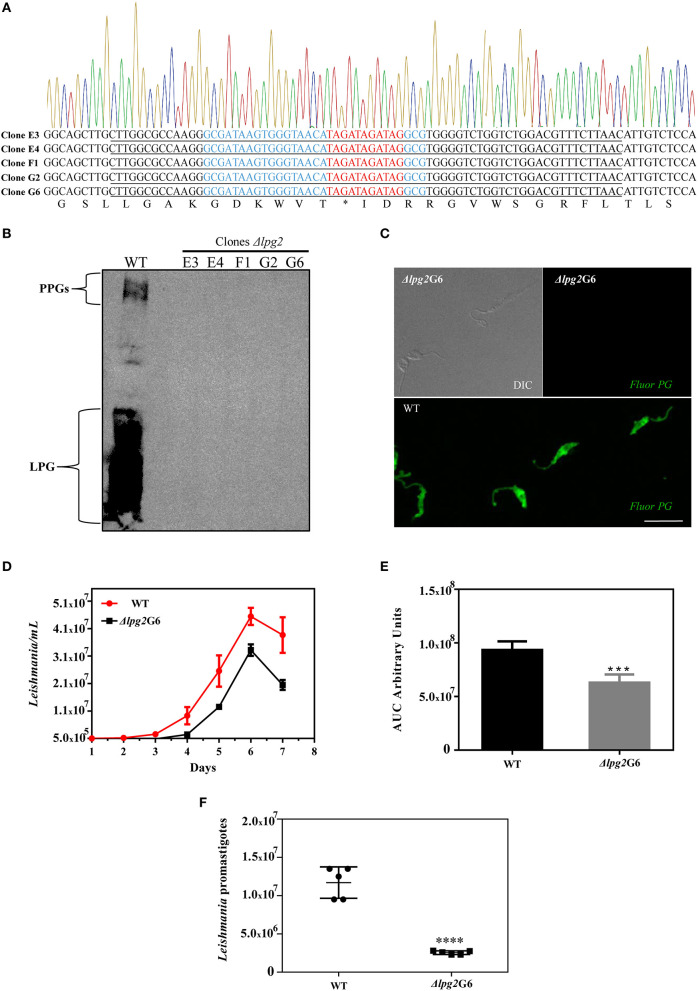
Molecular characterization of Δ*lpg2* and reduced virulence phenotype. **(A)** Chromatogram and translated sequence showing the region of the *LPG2* gene in which the precise insertion of a stop codon (denoted by an *) occurred by homologous recombination at the cleavage site of the Cas9 enzyme (nucleotides in red). The oligodonor sequence is underlined and the gRNA440 sequence is highlighted in blue. **(B)** Western blot analysis of the expression of LPG and PPGs in *L. infantum* promastigotes WT and Δ*lpg2* clones. **(C)** Confocal immunofluorescence analysis of WT and Δ*lpg2* (clone G6) parasites. Late log-phase promastigotes were adhered on Poly-L-Lysine-coated glass coverslips, fixed and incubated with the CA7AE antibody to visualize LPG and other Gal(β1,4)Man(α1-PO4) repeating unit-containing PGs (green). Differential interference contrast (DIC) for Δ*lpg2* is shown in the upper left panel. Scale bar: 10 μm. **(D)** Axenic growth curves of late log-phase promastigotes of *L. infantum* wild-type (WT) and clone G6 Δ*lpg2*, each point represents mean and SE. Data are representative of at least three independent assays and were collected in triplicate for each experimental condition. **(E)** Area under the curve (AUC) analysis of growth curves presented in **(D)**, ***p < 0.01. **(F)** Reduced virulence of Δ*lpg2* parasites in human neutrophils. Human neutrophils were infected with *L. infantum* Ba262 wild-type and Δ*lpg2* promastigotes for 3 h. Numbers of viable promastigotes shown after 24 h, with each point on the graph representing the cells from a health donor. Statistical differences were evaluated using the Student *t-*test, ****p < 0.001.

To examine the types of mutations created by the CRISPR-Cas9 system at the *LPG2*-targeting sites, genomic DNA was extracted from these non-agglutinating parasites after clonal isolation (clones denominated E3, E4, F1, G2, and G6). PCR was performed with primers designed to amplify the *LPG2* gene, followed by DNA sequencing of the targeted sites (Supplementary Figure 1B and Figure 3A). All five sequenced clones revealed the expected genome editing with oligodonor insertions, and no random insertions/deletions were observed within the sequenced region (nucleotides 15–870, Supplementary Figure 1C).

The loss of LPG expression in *L. infantum* Δ*lpg2* was demonstrated in promastigotes of all five isolated clones by Western blot, and by confocal immunofluorescence microscopy for clone G6 (Figures 3B,C). To better characterize these five knockout clones, growth curves were constructed and compared to wild-type parasites (Supplementary Figure 2A). This assay revealed no apparent differences in the growth profile of the five Δ*lpg2* clones. However, Area Under the Curve (AUC) analysis demonstrated a significant impairment in the growth rate of knockout parasites compared to WT. Our AUC analysis compared the average growth rate of all five knockout clones to WT (Supplementary Figures 2B,C), in addition to comparing one specific clone (G6) to WT (Figures 3D,E). The doubling time was calculated at 8.1 (G6) and 23.7 (WT). Despite differences in cell densities among Δ*lpg2* parasites, no significant delays were observed in comparison to wild-type parasites with respect to the time needed to reach stationary phase (Figure 3D and Supplementary Figure 2A). While it appeared that knockout and WT parasites seemed to differentiate into metacyclic forms at similar frequencies, since no apparent morphological differences were observed during growth phase, this warrants further investigation (e.g., through the analysis of ultrastructural and morphometric data or stage-specific gene expression).

Finally, the T7 Endonuclease I assay confirmed the absence of any non-edited versions of *LPG2* in the genomic DNA of clones (G6 and E4) as reveled by the absence of digested products following enzyme incubation (Supplementary Figure 2D). After selecting the Δ*lpg2* parasites, Cas9 protein expression was eliminated by removing the selection marker (G418) for the pLdCN vector, which was achieved after 5-6 passages (Supplementary Figure 2E). Together, these data indicate that the *LPG2* gene was successfully edited in the Δ*lpg2* mutants, resulting in the generation of a parasite deficient in LPG, as well as PG-containing molecules (e.g., PPGs).

### *Δlpg2* Parasites Exhibit Limited Survival in Neutrophils

To evaluate differences in parasite survival between WT and genome-edited parasites *in vitro*, human neutrophils were experimentally infected and incubated for 3 h. Neutrophils infected with Δ*lpg2* parasites exhibited an ~83% reduction in intracellular parasite load as estimated by parasite viability determined at 3 h after infection (Figure 3F).

## Discussion

We recently confirmed the involvement of LPG as a virulence factor in *L. infantum* through the use of parasites that had the *LPG1* gene knocked out (Lazaro-Souza et al., 2018). However, other phosphoglycan-containing molecules (PPGs, PGs and sAPs) may also play a role in parasite survival within host cells (Spath et al., 2003a; Gaur et al., 2009). Here we developed an *L. infantum* species parasite knocked-out for the *LPG2* gene, which did not express LPG or other PG-containing molecules (PPG, PGs, sAP).

Initially, we sought to obtain knockouts using the conventional method of homologous recombination involving resistance marker genes (Cruz et al., 1991). Despite some limitations, this method has been useful for single gene disruption and was shown to successfully knock out the *LPG2* gene in *L. donovani, L. Mexicana*, and *L. major* (Scianimanico et al., 1999; Ilg et al., 2001; Spath et al., 2003b). After performing two transformation rounds and selecting for the potential Δ*lpg2* parasites, the presence of an integral allele of the *LPG2* gene was still observed in genomic DNA, suggesting either that the homologous recombination process was inefficient, or that this gene was possibly duplicated in the *L. infantum* genome.

The homologous recombination gene disruption strategy was designed based on information present in an unplaced 19 kb genomic contig of *L. infantum* JPCM5 (GenBank accession CACT01000040.1), in which the *LPG2* gene was originally mapped (LinJ_34_4290). It is important to note that although this contig was described in association with chromosome 34, it was not physically mapped to this chromosome, which raised doubts regarding the consistency of this contig. Subsequent analysis in a new assembly of the *L. infantum* JPCM5 genome (Gonzalez-de la Fuente et al., 2017) indicated that this 19 Kb contig (including the *LPG2* gene) is duplicated in a tandem array located in chromosome 34. This new assembly, performed using PacBio and Illumina technology, was more robust, as evidenced by fewer gaps and increased identification of duplicate regions (tandem arrays). Interestingly, the duplication of the *LPG2* gene in the tandem array was not mentioned by these authors in their list of duplicated genes. More recently the *LPG2* duplication was spotted by a study that performed a comparative genomic and evolutionary analysis of the proteins involved in the biosynthesis of LPG in *Leishmania* (Azevedo et al., 2020).

The fact that the duplication of *LPG2* was not initially identified highlights limitations regarding the way *Leishmania* genomes are traditionally assembled, which usually entails the use of low-coverage whole-genome sequencing using short reads, with subsequent assembly performed using a reference genome obtained from a previously sequenced genome that can even belong to a different *Leishmania* species (Peacock et al., 2007). The presence of two copies of *LPG2* in *L. infantum* limited our ability to perform genetic manipulation via homologous recombination in this parasite species, which emphasizes the need to improve the overall quality of *Leishmania* genomes, i.e., completeness and contiguity, preferentially through the use of recently employed hybrid sequencing strategies (short and long reads) (Gonzalez-de la Fuente et al., 2017, 2019; Lypaczewski et al., 2018). In the end, we overcame this limitation through the use of the CRISPR/Cas9 system for genome editing.

The CRISPR/Cas9 system used here proved more flexible in producing *Leishmania* knockouts without the use of genetic resistance markers for selection, thereby allowing for more direct comparisons with WT parasites. In addition, the use of oligodonors containing stop codons permitted more specific editing of the target gene as opposed to random insertions/deletions (Zhang et al., 2017; Zhang and Matlashewski, 2019).

Previous studies found no evidence of Cas9 toxicity in *Leishmania mexicana*, since promastigote forms grew and reached stationary phase at similar rates and densities as wild-type cells (Beneke et al., 2017; Martel et al., 2017). However, others have reported some reduced phenotypic growth in the late stages of culturing (Ishemgulova et al., 2018). The reasons behind these divergent results remain unclear and could rely on the way that this expression is conducted (episomal vs. genomic integration) and also the window of time analyzed, but further study will be necessary.

As the generated *L. infantum* strain expressing Cas9-gRNA presented delayed growth, we speculate that this could be due to the action of Cas9-gRNA and the low efficiency of mechanisms involved in repairing double-strand breaks in *Leishmania*, which may lead to cell cycle arrest and even cell death, as demonstrated by (Zhang and Matlashewski, 2019). Importantly, future studies must address this issue, which remains unclear: Does the expression of Cas9 *per se* negatively affect *Leishmania* growth, or this is a result of Cas9 expression together with a *Leishmania*-DNA-targeting-gRNA?

For the selection and clonal isolation of the Δ*lpg2* parasites, we initially used the CA7AE monoclonal antibody that recognizes the Gal(β1,4)Man(α1-PO4) repeat region of the LPG molecule (Descoteaux et al., 1998). However, after five rounds of agglutination, there was reason to believe that wild-type parasites could still be present in knockout cultures, due to the fact that it was not possible to completely eliminate wild-type parasites in control cultures. These results highlight a limitation associated with the use of this antibody to select Δ*lpg2* parasites in an *L. infantum* model without the additional presence of a resistance gene marker. To overcome this limitation, we chose to employ a lectin (RCA 120), which recognizes terminal β-galactose residues, that was shown to be cytotoxic to wild-type *L. donovani* and *L. major* parasites, but did not affect parasites that do not present LPG and other PG-containing molecules (King and Turco, 1988; Cappai et al., 1994; Opat et al., 1996). After two rounds of agglutination, the use of this lectin resulted in wild-type parasite death and complete elimination. Despite the absence of resistance markers to select knockout parasites, the CRISPR/Cas9 system, coupled to oligodonors, demonstrated high efficiency in target gene editing, as evidenced by the analysis of sequencing data from five of the generated clones, which all presented the expected result at the site of Cas9 cleavage. In addition, Western blotting and confocal microscopy demonstrated the complete elimination of PG-repeating units in both LPG and other PG-containing molecules, which is consistent with the literature (Descoteaux et al., 1995; Dermine et al., 2000; Spath et al., 2003b; Lazaro-Souza et al., 2018). However, it will be important for future experimental design to quantitatively investigate system efficiency by determining how many null mutants are generated as a fraction of oligodonor-transfected parasites.

After selecting the Δ*lpg2* parasites, Cas9 protein expression was eliminated by removing the selection marker (G418) for the pLdCN vector, which was achieved after 5–6 passages (Supplementary Figure 2E). No differences in phenotype or growth rates were observed among the five isolated clones, which suggest that, if present, off-target effects had little impact. Although low cell density was observed when the Δ*lpg2* parasites reached stationary phase, the time required to reach this phase was not significantly delayed. This finding stands in contrast to delays seen in achieving stationary phase when knockout parasites are obtained by homologous recombination using resistance markers, which likely occurs due to the presence of antibiotics in culture medium (Lazaro-Souza et al., 2018). In addition, we did not observe any significant morphological or ultrastructural changes, indicating that the deletion of the *LPG2* gene did not interfere with the cellular biology of *L. infantum* promastigotes.

In an attempt to obtain insights regarding the role of LPG and PG-containing molecules in cells present at initial stages of infection, human neutrophils were infected for 3 h with *L. infantum* Δ*lpg2*, revealing a significant reduction in infection rate compared to WT. Neutrophils, essential innate immune system cells, are rapidly recruited to the site of inflammation and have been described as playing a critical role in *Leishmania* infection (Ribeiro-Gomes et al., 2004; Hurrell et al., 2016). In addition, LPG was shown to be important to the induction of autophagy in neutrophils using a model of *L. donovani* infection (Pitale et al., 2019). However, our findings in this cell type must be considered preliminary, as it will be necessary to investigate additional time points and the underling mechanisms. Future studies should also extend the scope of investigation to include macrophages.

In sum, the results presented herein confirm the unexpected duplication of the *LPG2* gene in the *L. infantum* genome. The successful inactivation of both copies of this gene using the CRISPR/Cas9 system highlights the potential of using these KO parasites to assess signal transduction pathways elicited by PG-containing molecules from a variety of *Leishmania* species in different types of immune cells. In addition, the technique demonstrated herein of editing the *LPG2* gene using CRISPR exemplifies the prospects of inactivating other virulence factors sequentially, which could pave the way for new vaccine development.

## Data Availability Statement

The datasets generated for this study are available on request to the corresponding author.

## Ethics Statement

The studies involving human participants were reviewed and approved by Institutional Review Board of Human Ethical Research Committee of Fundação Oswaldo Cruz-Bahia. Written informed consent for participation was not required for this study in accordance with the national legislation and the institutional requirements.

## Author Contributions

FJ-S, PR, AD, JL, VB, and LF conceived and designed the study and contributed to data analysis. FJ-S, JL, JL-S, and LF performed the experiments. FJ-S, JL, PR, AD, JL-S, VB, and LF wrote and critically revised the manuscript. All authors have read and approved the final version of this manuscript.

## Conflict of Interest

The authors declare that the research was conducted in the absence of any commercial or financial relationships that could be construed as a potential conflict of interest.

## Supplementary Material

Supplementary Figure 1 Detail of *LPG2* gene locus indicating gRNA targeting sites. **(A)** Schematic representation of the wild-type alleles of the *LPG2* gene (magenta box) present in a duplicated tandem array. **(B)** Schematic representation showing the insertion of a stop codon in *LPG2* alleles following Cas9 genome editing (white line interrupting the magenta box). **(C)** *LPG2* sequence depicting: gRNA440 sequence in (blue), oligonucleotide donor sequence (underlined) and inserted stop codons (red). Arrows represents primers used for *LPG2* sequence and Δ*lpg2* characterization.

Supplementary Figure 2 Characterization of Δ*lpg2* clones. **(A)** Growth curve of wild-type (WT) *L. infantum* promastigotes and five different Δ*lpg2* clones. **(B)** Analysis of growth curve of *L. infantum* wild-type (WT) promastigotes and mean growth rates of all five different Δ*lpg2* clones **(C)** Area under the curve (AUC) of growth curve presented in **(B)**. **(D)** T7 Endonuclease I assay using genomic DNA from *L. infantum* wild-type (WT) promastigotes and two clones (E4 and G6) of *L. infantum* Δ*lpg2*. A positive control containing equal amounts of WT and KO DNA demonstrates the presence of two bands following digestion. **(E)** Western blot analysis of *L. infantum* promastigotes (clone G6) demonstrating the loss of the pLdCN vector after 5–6 passages in the absence of the G418 marker.

Supplementary Table 1 Oligonucleotides sequences used to assemble the fragments pLPG2-Hyg and pLPG2-Neo for gene targeting of LPG2 by homologous recombination and CRISPR/Cas9.
