# Supplementary material for: LPG2 Gene Duplication in Leishmania infantum: A Case for CRISPR-Cas9 Gene Editing
Source: Front Cell Infect Microbiol. 2020 Aug 13;10:408. doi: 10.3389/fcimb.2020.00408 (PMC7438834; doi:10.3389/fcimb.2020.00408)
Supplement: Supplementary Figure 1 — Detail of LPG2 gene locus indicating gRNA targeting sites. (A) Schematic representation of the wild-type alleles of the LPG2 gene (magenta box) present in a duplicated tandem array. (B) Schematic representation showing the insertion of a stop codon in LPG2 alleles following Cas9 genome editing (white line interrupting the magenta box). (C) LPG2 sequence depicting: gRNA440 sequence in (blue), oligonucleotide donor sequence (underlined) and inserted stop codons (red). Arrows represents primers used for LPG2 sequence and Δlpg2 characterization. [file Presentation_1.PPTX]

## Slide 1
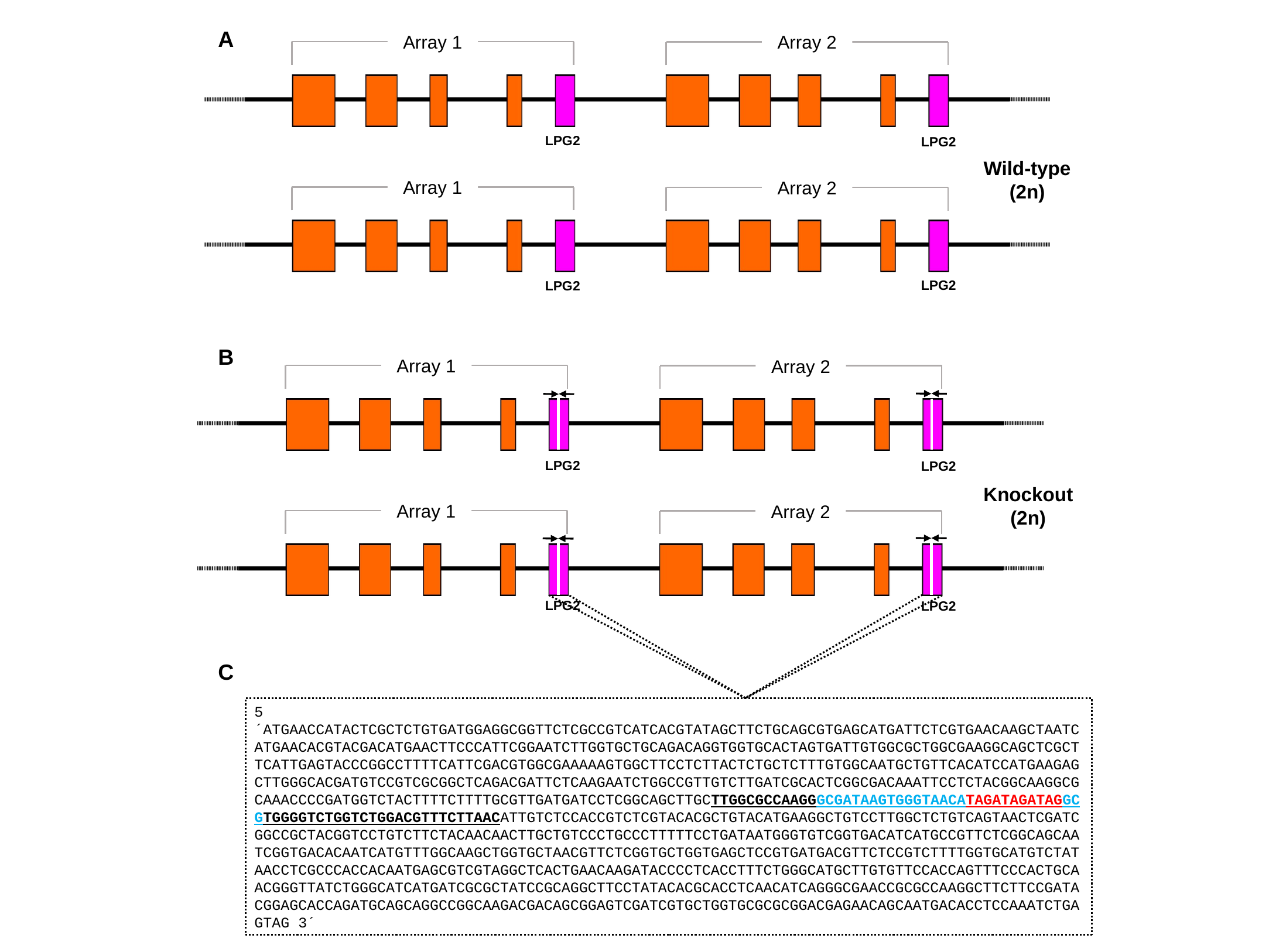

A
Array 1
Array 2
LPG2
LPG2
Wild-type
(2n)
Array 1
Array 2
LPG2
LPG2
B
Array 1
Array 2
LPG2
LPG2
Knockout
(2n)
Array 1
Array 2
LPG2
LPG2
C
5´ATGAACCATACTCGCTCTGTGATGGAGGCGGTTCTCGCCGTCATCACGTATAGCTTCTGCAGCGTGAGCATGATTCTCGTGAACAAGCTAATCATGAACACGTACGACATGAACTTCCCATTCGGAATCTTGGTGCTGCAGACAGGTGGTGCACTAGTGATTGTGGCGCTGGCGAAGGCAGCTCGCTTCATTGAGTACCCGGCCTTTTCATTCGACGTGGCGAAAAAGTGGCTTCCTCTTACTCTGCTCTTTGTGGCAATGCTGTTCACATCCATGAAGAGCTTGGGCACGATGTCCGTCGCGGCTCAGACGATTCTCAAGAATCTGGCCGTTGTCTTGATCGCACTCGGCGACAAATTCCTCTACGGCAAGGCGCAAACCCCGATGGTCTACTTTTCTTTTGCGTTGATGATCCTCGGCAGCTTGCTTGGCGCCAAGGGCGATAAGTGGGTAACATAGATAGATAGGCGTGGGGTCTGGTCTGGACGTTTCTTAACATTGTCTCCACCGTCTCGTACACGCTGTACATGAAGGCTGTCCTTGGCTCTGTCAGTAACTCGATCGGCCGCTACGGTCCTGTCTTCTACAACAACTTGCTGTCCCTGCCCTTTTTCCTGATAATGGGTGTCGGTGACATCATGCCGTTCTCGGCAGCAATCGGTGACACAATCATGTTTGGCAAGCTGGTGCTAACGTTCTCGGTGCTGGTGAGCTCCGTGATGACGTTCTCCGTCTTTTGGTGCATGTCTATAACCTCGCCCACCACAATGAGCGTCGTAGGCTCACTGAACAAGATACCCCTCACCTTTCTGGGCATGCTTGTGTTCCACCAGTTTCCCACTGCAACGGGTTATCTGGGCATCATGATCGCGCTATCCGCAGGCTTCCTATACACGCACCTCAACATCAGGGCGAACCGCGCCAAGGCTTCTTCCGATACGGAGCACCAGATGCAGCAGGCCGGCAAGACGACAGCGGAGTCGATCGTGCTGGTGCGCGCGGACGAGAACAGCAATGACACCTCCAAATCTGAGTAG 3´
