# Supplementary figures and images for: LPG2 Gene Duplication in Leishmania infantum: A Case for CRISPR-Cas9 Gene Editing
Source: Front Cell Infect Microbiol. 2020 Aug 13;10:408. doi: 10.3389/fcimb.2020.00408 (PMC7438834; doi:10.3389/fcimb.2020.00408)

## Slide 1
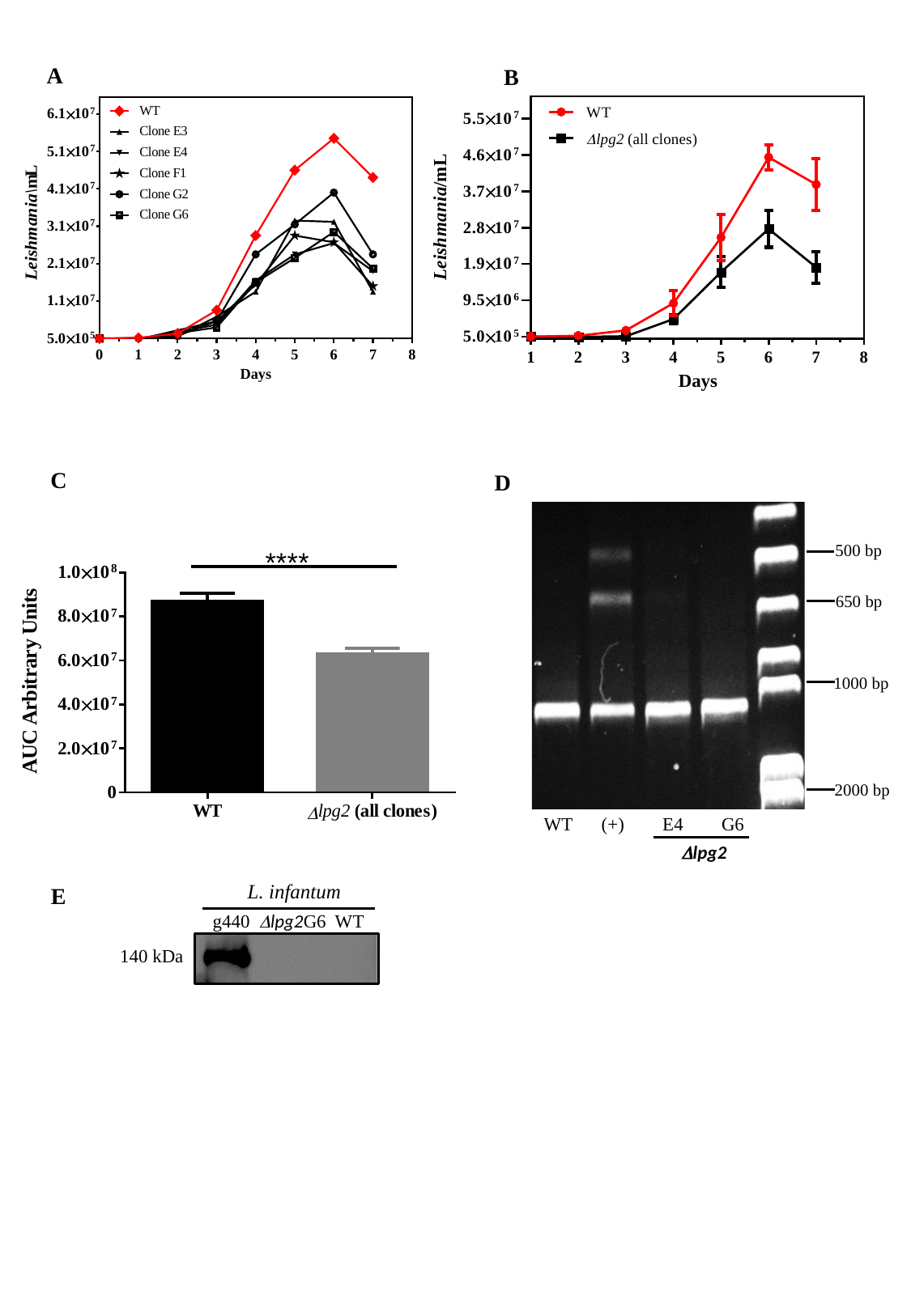

A
B
C
D
WT (+) E4 G6
2000 bp
1000 bp
650 bp
500 bp
Δlpg2
L. infantum
E
 g440 Δlpg2G6 WT
140 kDa

Supplement: Supplementary Figure 2 — Characterization of Δlpg2 clones. (A) Growth curve of wild-type (WT) L. infantum promastigotes and five different Δlpg2 clones. (B) Analysis of growth curve of L. infantum wild-type (WT) promastigotes and mean growth rates of all five different Δlpg2 clones (C) Area under the curve (AUC) of growth curve presented in (B). (D) T7 Endonuclease I assay using genomic DNA from L. infantum wild-type (WT) promastigotes and two clones (E4 and G6) of L. infantum Δlpg2. A positive control containing equal amounts of WT and KO DNA demonstrates the presence of two bands following digestion. (E) Western blot analysis of L. infantum promastigotes (clone G6) demonstrating the loss of the pLdCN vector after 5–6 passages in the absence of the G418 marker. [file Presentation_2.PPTX]
