# Supplementary material for: LPG2 Gene Duplication in Leishmania infantum: A Case for CRISPR-Cas9 Gene Editing
Source: Front Cell Infect Microbiol. 2020 Aug 13;10:408. doi: 10.3389/fcimb.2020.00408 (PMC7438834; doi:10.3389/fcimb.2020.00408)
Supplement: Supplementary Table 1 — Oligonucleotides sequences used to assemble the fragments pLPG2-Hyg and pLPG2-Neo for gene targeting of LPG2 by homologous recombination and CRISPR/Cas9. [file Table_1.DOCX]

Supplementary Table 1- Oligonucleotides sequences used to assemble the fragments p*LPG2*-Hyg and p*LPG2*-Neo for gene targeting of LPG2 by homologous recombination and CRISPR/Cas9.

| Homologous Recombination | | |
| --- | --- | --- |
| Fragment | **Forward Primer (5’ – 3’)**  **Reverse Primer (5’ – 3’)** | **Restriction sites** |
| 5UTR’-LPG2-3’UTR | GATATCGCTTCCATCTGAAATGTGCTG  TCGCATGGTGCGATGCAGCTGTAG | - |
| LPG2 5’UTR | CTGgagctcgatatcgcttccatctgA  CATGGTACCGGCAAATGCTGATGCAATCC | EcoRV/SacI  KpnI |
| LPG2 3’UTR | GTGtctagaAGTAGTCACTGCTGTTAGCAG  CAGaagcttAtggtgcgatgcagctgtag | XbaI  HindIII |
| LPG2 | GTAAAGCTTATGAACCATACTCGCTCTG  GTAAAGCTTCTACTCAGATTT*GGAGGT*G | HindIII  HindIII |
| Hyg | GCCGGATCCATGAAAAAGCCTGAACTCA  GTCGGATCCCTATTCCTTTGCCCTCGG | BamHI  BamHI |
| Neo | AAGGGATCCATGGGATCGGCCATTGAA  CTGGGATCCTCAGAAGAACTCGTCAAGAA | BamHI  BamHI |
| CRISPR/Cas9 | | |
| gRNA440 | TTGTGCGATAAGTGGGTAACAGCG  AAACCGCTGTTACCCACTTATCGC | - |
| gRNA516 | TTGTGTACAGCGTGTACGAGACGG  AAACCCCTCTGCTACACGCTGTAC | - |
| OD440 | 5’CTTGGCGCCAAGGGCGATAAGTGGGTAACATAGATAGATAGGCGTGGGGTCTGGTCTGGACGTTTCTTAAC 3’ | - |
| OD516 | 5´AAG GACAGCCTTCATGTACAGCGTGTACGACTATC TATCTAGACGGTGGAGACAATGTTAAGAAACGTCCA 3’ | - |
|  |  | - |

Red – STOP codons
